# Benthic foraminiferal diversity in the Arabian Gulf: spatial patterns in a basin-wide assessment

**DOI:** 10.1371/journal.pone.0327033

**Published:** 2025-07-10

**Authors:** Abduljamiu Olalekan Amao, Khalid Al-Ramadan, Michael Kaminski, Fabrizio Frontalini

**Affiliations:** 1 Center for Integrative Petroleum Research, King Fahd University of Petroleum and Minerals, Dhahran, Saudi Arabia; 2 Geosciences Department, King Fahd University of Petroleum and Minerals, Dhahran, Saudi Arabia; 3 Department of Pure and Applied Sciences, Urbino University, Urbino, Italy; University of Palermo: Universita degli Studi di Palermo, ITALY

## Abstract

Benthic foraminifera play crucial roles in marine ecosystems and serve as valuable bioindicators of ecological conditions and environmental changes. Despite their importance, comprehensive basin-wide assessments of their diversity patterns remain scarce, particularly in complex environments like the Arabian Gulf. This study reveals the variation of benthic foraminiferal diversity within the gulf and provides insights into its distribution patterns and relationships with environmental gradients. We compiled a comprehensive dataset of benthic foraminiferal occurrences from published literature and public databases, encompassing more than 492 species from nine orders, 39 superfamilies, 89 families, and 150 genera. Using an ensemble of species distribution models, we modelled the spatial patterns of individual species and stack these predictions to estimate foraminiferal species richness across the basin. We documented a pronounced north-south diversity gradient that differs from typical latitudinal patterns observed in larger marine systems. Our methodological framework identifies bathymetry and dissolved oxygen as primary drivers of foraminiferal distributions when averaged across all species, with significant influence from iron concentration and salinity. However, individual species showed diverse environmental responses, with variables of lower mean importance often exerting primary control on specific taxa, highlighting the ecological specialization that enables such high diversity in this extreme environment. The east-west diversity gradient reveals the impact of basin-scale circulation patterns on foraminiferal assemblage composition, a phenomenon relevant to other semi-enclosed seas worldwide. The models show high performance (mean AUC > 0.94, TSS > 0.8, Kappa > 0.82), demonstrating the potential of this approach in capturing complex species-environment relationships. Additionally, model predictions align well with known foraminiferal distributions and diversity patterns reported in previous studies across the gulf. This study provides the first basin-wide assessment of benthic foraminiferal diversity in the Arabian Gulf, revealing complex spatial patterns and environmental relationships. Most significantly, our delineation of species-specific ecological niches provides a valuable framework for forecasting foraminiferal responses to climate-driven environmental changes, particularly thermal stress, which our models identify as more influential in its extreme rather than mean values.

## Introduction

Understanding the spatial distribution of species is a fundamental goal in ecology and biogeography, providing insights into ecosystem functioning, biodiversity patterns, and environmental change [1,2]. This knowledge extends beyond academic interest, informing our ability to predict ecological responses to anthropogenic impacts across diverse environments [3]. Benthic foraminifera, single-celled protists with calcium carbonate or agglutinated tests, represent an ideal model system for investigating spatial ecology due to their ubiquity, high diversity, and exceptional fossil record spanning over 500 million years [4–6]. These organisms contribute substantially to global carbonate production, participate in marine carbon and nutrient cycling, and remain among the most valuable proxies for paleoenvironmental reconstructions across geological time scales [7–9]. The ecological and paleoceanographic significance of benthic foraminifera has driven decades of research into their spatial distributions [10–14]. Early investigations relied primarily on field sampling to document presence and abundance patterns, generating detailed but geographically constrained datasets. These foundational studies established our understanding of foraminiferal ecology, enabling the creation of distribution maps, defining species’ environmental preferences, and documenting assemblage composition across environmental gradients [15–17]. However, the localized nature of these investigations has typically limited their applicability to broader spatial scales, highlighting the need for more integrative, basin-wide approaches that can bridge local and regional patterns [18,19].

Several important basin-wide approaches have been successfully implemented in the past, such as Jorissen’s comprehensive work in the Adriatic Sea [20], Culver and Buzas’ extensive studies along the Atlantic continental margin [21], Pfleger’s pioneering research in the Gulf of Mexico [22], and Murray’s detailed analyses in the Arabian Gulf [23], the integration of these datasets with modern computational tools now offers new opportunities to examine foraminiferal distributions at unprecedented resolution. Recent advances in species distribution modelling provide complementary approaches that can efficiently synthesize extensive data from multiple studies, further enhancing our ability to bridge local and regional patterns and potentially reveal ecological insights that might be less apparent with traditional methods [18,19]. The distribution of benthic foraminifera reflects a complex interplay of environmental factors operating across multiple spatial and temporal scales [10,24]. At basin and regional scales, temperature, salinity, depth, oxygen, carbonate saturation state, and productivity gradients shape distinct habitat preferences from coastal marshes to the deep ocean [25–27]. These factors generate biogeographic patterns that reflect both historical evolutionary processes and contemporary environmental conditions [28]. At finer scales, substrate characteristics, hydrodynamic regimes, oxygen availability, organic matter flux, and disturbance events further modulate microhabitat structure and assemblage composition [29–31]. This multi-scale complexity presents significant challenges for predicting foraminiferal distributions and diversity patterns, particularly in environmentally extreme and heterogeneous settings where traditional ecological models may not be applied [32].

Recent advances in data integration and remote sensing technologies have transformed ecological research, enabling the testing and development of ecological theories at unprecedented spatial scales [33,34]. These technological developments have facilitated the collection and integration of extensive environmental and biological census data, creating new opportunities for understanding species distributions beyond individual study sites. Modern foraminiferal ecologists have increasingly utilized species distribution models (SDMs) to estimate spatiotemporal patterns of species occurrence, relating distributional data to environmental predictors [35,36]. These models operate within ecological niche theory, which defines the set of environmental conditions permitting species persistence [37,38]. By leveraging machine learning algorithms and statistical approaches, SDMs can capture complex, non-linear relationships between species and their environment, providing powerful tools for both fundamental ecological research and applied environmental assessment [39]. Methodological innovations have also extended beyond single-species approaches to community-level SDMs, such as stacked species distribution models (SSDMs), which combine individual species models to predict community properties like species richness and composition [40,41]. This advancement allows researchers to transcend species-specific predictions and explore emergent patterns at the assemblage level. When appropriately implemented, SSDMs perform comparably to macroecological models in predicting biodiversity patterns across unsampled regions [42,43]. This capability proves particularly valuable for studying benthic foraminifera, where the high diversity (>10,000 described species) and complex ecological interactions necessitate community-level approaches to understand distribution patterns and diversity gradients across heterogeneous environmental settings. SDMs have been widely applied to terrestrial systems, their application to marine organisms remains relatively limited, creating a significant knowledge gap in our understanding of marine biogeography and applied community ecology [44]. Semi-enclosed basins like the Arabian Gulf offer ideal natural laboratories for testing SDM approaches in marine systems, as they contain compressed environmental gradients within defined geographic boundaries, facilitating the identification of species-environment relationships that may be less apparent in open ocean systems [42,45].

The Arabian Gulf, characterized by extreme temperature and salinity regimes that exceed global averages, provides an exceptional setting for determining environmental thresholds and ecological tolerances in foraminiferal communities [40,41]. Understanding these tolerance thresholds has direct implications for predicting biotic responses to climate change in other marine systems globally [46]. Research on benthic foraminifera in the Gulf has significantly progressed since Murray’s pioneering studies [23,47–52] in the Abu Dhabi region, which identified 58 species and established the influence of extreme environmental conditions on foraminiferal assemblages. Subsequent investigations expanded our understanding of basin-wide distributions, with Lutze [53–55] documenting assemblages along the Iranian coast, Haake [56] analyzing miliolid distributions in offshore sediments, and Cherif et al. [57] characterizing 98 species from the central Gulf and their environmental relationships. In one recent comprehensive synthesis [18], six decades of studies documenting 753 species from 236 genera across the Gulf were integrated. The analysis identified distinct ecological zones with clear spatial patterns in assemblage composition. Porcelaneous (44.6%) and hyaline taxa (42.9%) dominated the assemblages, while agglutinated forms (12.4%) showed limited distribution, attributed to high salinity and restricted terrigenous input. Despite this extensive documentation, taxonomic uncertainties persist, with many species still described in open nomenclature. Additionally, certain areas of the basin remain undersampled, particularly in the southwestern region, creating significant knowledge gaps in our understanding of the Gulf’s foraminiferal community.

In this study, we map the species richness of benthic foraminifera in the Arabian Gulf using SSDMs built on ensemble modeling techniques. This approach integrates data from numerous local investigations, offering a basin-wide perspective on foraminiferal diversity patterns [42,45] and filling in the gaps in unsampled areas. By building robust, high-resolution models of individual species distributions and environmental preferences, we establish relationships between foraminiferal diversity and environmental parameters that can inform broader ecological theory regarding biodiversity patterns in extreme environments [58]. The methodological framework developed here can enhance understanding and prediction of foraminiferal distribution patterns across other semi-enclosed seas globally [59,60].

### Oceanographic setting of the Arabian Gulf

**Fig 1:**
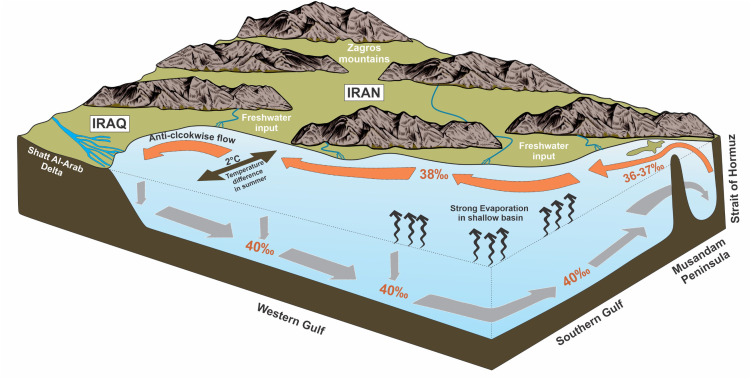
Map showing the main oceanographic features of the Arabian Gulf, including general circulation pattern, water mass exchange through the Strait of Hormuz, major river inputs, and temperature and salinity gradients.

The Arabian Gulf is characterized by a counterclockwise circulation pattern primarily driven by density gradients, wind forcing, and freshwater inputs [18,61,62]. Water exchange with the Indian Ocean occurs through the Strait of Hormuz, where less saline surface waters (36.5‰) flow into the Gulf, while denser, more saline bottom waters exit as a subsurface outflow [62,63] (Fig 1). The Gulf experiences significant seasonal temperature variations, with offshore water temperatures ranging from 20–32°C [64] and western Gulf temperatures ranging from 16–36°C [65]. In shallow lagoonal areas, temperatures can fluctuate between 15–40°C [66,67]. Due to its shallow bathymetry (mean depth 35 m), most of the Gulf lies within the photic zone, with few exceptions along the deeper Iranian coast [10].

Water column structure in the Arabian Gulf varies both spatially and seasonally, producing diverse microhabitats for benthic communities. The shallow southern and western margins (<20 m depth) remain relatively well-mixed throughout the year due to wind action and limited depth, creating environments where surface conditions strongly influence benthic habitats [61]. In contrast, the deeper central and eastern portions develop pronounced seasonal stratification. During summer months, a thermocline forms at approximately 20–30 m depth, particularly along the Iranian coast where depths reach 60–80 m, generating distinct layers with limited vertical exchange [68].

Salinity distribution exhibits distinct spatial patterns across the basin. Surface waters entering through the Strait of Hormuz have a salinity of approximately 37‰, which increases to about 40‰ in the central Gulf [61]. Along the shallow southern and western coasts, particularly in lagoons and embayments southeast of Qatar, salinity can exceed 50‰ [23]. In contrast, the northern Gulf near the Shatt Al-Arab delta experiences lower salinities (approximately 36‰) due to freshwater input and mixing [63]. The hydrodynamic regime of the Gulf, is influenced by complex tidal patterns that vary from diurnal to semi-diurnal and mixed types [61]. The predominant northwesterly Shamal winds significantly influence surface circulation and contribute to aeolian sediment input throughout the year [61]. These winds, combined with the shallow bathymetry, facilitate vertical mixing in much of the basin. The average residence time of water in the Gulf is approximately 2–5 years [69], which amplifies the effects of local environmental stressors and contributes to the Gulf’s sensitivity to both natural and anthropogenic changes.

While generally oligotrophic, the Gulf experiences seasonal phytoplankton blooms, particularly in winter (December-February) when cooler temperatures reduce stratification [70]. These blooms affect organic matter flux to the seafloor, a critical factor for benthic foraminiferal communities. Seasonal coastal upwelling occurs along parts of the Iranian coast, driven by the interaction between the counterclockwise circulation and bathymetric features [71]. These zones of upwelled, nutrient-rich water create localized productivity hotspots that influence benthic communities.

Freshwater input primarily occurs through the Shatt Al-Arab delta in the northwest and several rivers originating from the Zagros mountains along the Iranian coast [66,72]. These rivers, including Arvand Rud (Shatt Al-Arab), Gamasb, Karun, Jarahi, Zohreh, Dalaki, Mend, Shur, Minab, Mehran, and Naband, deliver significant volumes of freshwater and terrigenous sediment to the Gulf, creating localized reductions in salinity and increases in turbidity along the northern and eastern margins.

## Methodology

### Data collection and preparation

**Fig 2:**
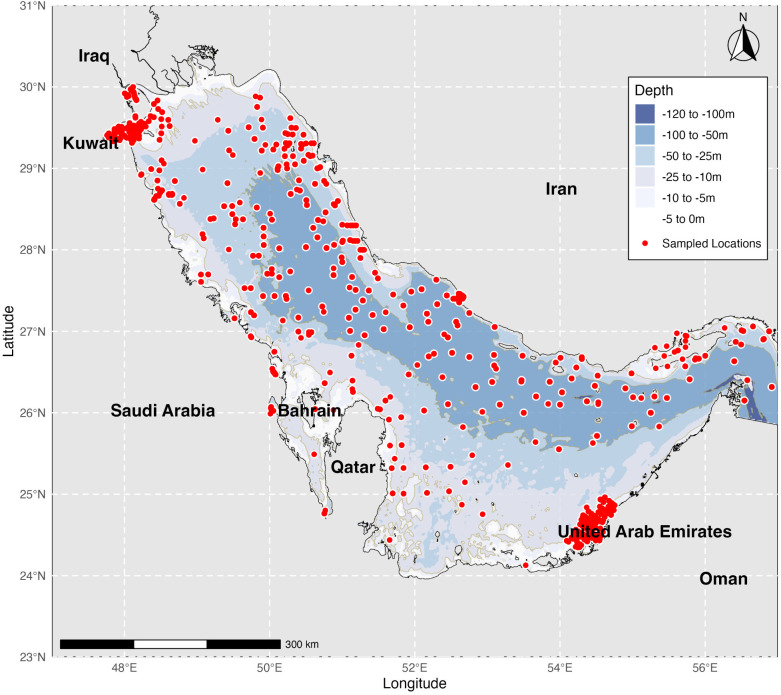
Bathymetric map of the Arabian Gulf showing sampling locations of benthic foraminiferal studies. The color gradient represents water depth, ranging from −120 m (dark blue) to 0 m (light grey). Red dots indicate sampling sites showing the spatial distribution of data collection across various depth zones and geographical areas.

To map benthic foraminiferal diversity in the Arabian Gulf, we compiled a dataset of species occurrences from published literature [18]. This dataset represents a synthesis of foraminiferal research in the region, encompassing various habitats and environmental conditions. Each occurrence records documented species name, geographic coordinates, depth, and associated metadata, providing a foundation for our analyses (Fig 2). Environmental data, important for understanding the abiotic factors shaping foraminiferal distributions, were obtained from multiple sources. We used the Bio-ORACLE database [73], which offers high-resolution marine environmental data layers designed for species distribution modelling. Additionally, we incorporated bathymetric data from the General Bathymetric Chart of the Oceans [74], ensuring a representation of the Gulf’s seafloor topography. These datasets provided information on key environmental variables known to influence foraminiferal distributions, including temperature, salinity, chlorophyll-*a* concentration, dissolved oxygen, and bathymetry [6,10].

### Data preprocessing

Data preprocessing and handling were conducted using the R programming language [75]. We implemented a data cleaning protocol to address common issues in occurrence data. This included removing duplicate records, correcting taxonomic inconsistencies, addressing synonyms, and filtering out records with insufficient spatial precision. To ensure taxonomic consistency across our dataset, species names were standardized against the World Register of Marine Species (WoRMS). Given the presence-only nature of our occurrence data, we employed a spatially stratified random sampling approach to generate pseudo-absences. The number of pseudo-absences was selected based on recommendations from recent literature for each modelling algorithm, balancing the need for robust model training with the avoidance of artificial patterns [76,77]. Environmental layers underwent processing to ensure spatial and temporal consistency. All layers were resampled to a common resolution of 1 km² using bilinear interpolation, balancing the native resolution of environmental data and the spatial accuracy of our occurrence records. The layers were then cropped to the extent of the Arabian Gulf. To address potential multicollinearity among environmental predictors, we calculated Pearson correlation coefficients between all pairs of variables. In cases of high correlation, we retained the variable with the most direct ecological relevance to foraminiferal distributions, guided by expert knowledge and literature review [10,24].

### Species distribution modelling

We employed a SSDM approach to predict the spatial patterns of benthic foraminiferal diversity in the Arabian Gulf. This method involves building individual SDM for each species and then combining these models to estimate community-level properties such as species richness. Individual SDMs were constructed using the SSDM package in R [78], which facilitates the implementation of multiple modelling algorithms. We utilized a range of algorithms known for their performance in SDM applications: Classification Tree Analysis (CTA), Generalized Boosted Models (GBM), Generalized Linear Models (GLM), Random Forests (RF), Support Vector Machines (SVM), Maximum Entropy (MaxEnt), Generalized Additive Models (GAM), Multivariate Adaptive Regression Splines (MARS), and Artificial Neural Networks (ANN). Recognizing that no single model consistently outperforms others across different species and environments [79], we adopted an ensemble modelling approach. This method combines predictions from multiple models, potentially improving predictive accuracy, reducing model-specific biases, quantifying uncertainty, and increasing overall robustness [80,81]. To optimize model performance and manage computational demands, we first conducted a subset analysis on fifty randomly selected species. This subset was chosen to ensure sufficient occurrence data for model calibration and evaluation. We systematically evaluated various combinations of modelling algorithms, iterating from ensembles of three algorithms up to five, to determine the optimal combination for our dataset.

Model performance was evaluated using a suite of metrics, including the Area Under the Receiver Operating Characteristic Curve (AUC), True Skill Statistic (TSS), Cohen’s Kappa, specificity, sensitivity, Jaccard index, and prediction success. We implemented a modelling protocol that included k-fold cross-validation (k = 20) to assess model stability and prevent overfitting. The TSS was used to determine the optimal threshold for converting continuous probability maps into binary presence-absence predictions. Each model was evaluated with multiple replicates to account for variability in model outcomes. We also assessed the relative importance of environmental variables using a permutation approach, randomly altering the values of each variable and measuring the resulting decrease in model performance. This analysis provides insights into the key drivers of foraminiferal distributions in the Gulf. The top five best-performing algorithm combination, as determined by our evaluation, was then applied to the full dataset of all documented foraminiferal species in the Gulf.

### Diversity mapping and analysis

Species richness maps were created by stacking the individual SDMs, following the method described by Calabrese et al. [41]. We employed a threshold approach to convert continuous probability maps into binary presence-absence predictions, with the threshold value determined by maximizing the sum of sensitivity and specificity. The final species richness map was produced by summing these binary presence-absence maps across all modelled species, providing a view of foraminiferal diversity across the Gulf. To account for prediction uncertainty, we generated uncertainty maps based on the variability in predictions across the ensemble of models [80]. Additionally, we created endemism maps to highlight areas hosting unique and rare species [82]. All spatial analyses and visualizations were performed using the *raster* [83] and *tidyverse* [84] packages in R.

### Model validation and limitations

To validate our models, we compared predicted species richness patterns with known diversity hotspots and biogeographic patterns described in the literature for the region [18,85]. This comparison allowed us to assess the ecological realism of our predictions and identify areas of agreement and divergence with existing knowledge. We also conducted sensitivity analyses to assess the impact of key modelling decisions, such as the choice of environmental variables and modelling algorithms, on our results [86]. While our approach represents an advance in mapping foraminiferal diversity at a basin-wide scale, we acknowledge several limitations inherent in SDM. These include potential sampling biases in the occurrence data, the assumption of equilibrium between species and their environment, and the inability to account for biotic interactions and dispersal limitations [87,88]. We discuss these limitations and their implications for interpreting our results in the discussion section, providing a view of the strengths and weaknesses of our approach.

## Results

### Environmental characteristics of the Arabian Gulf

Remote sensing and modelling data revealed complex environmental gradients across the Gulf, characterized by shallow bathymetry and extreme conditions. Bathymetry (Fig 2), a key driver of environmental conditions, ranged from −127.00 m to −2.00 cm (mean = −34.80 m, SD = 23.13 m-). Sea surface temperatures exhibited significant spatiotemporal variation (annual mean range: 24.23–29.45°C; mean = 26.86°C, SD = 1.00°C), with extreme seasonal fluctuations (13.09–37.60°C). While surface temperature measurements are reported here, our analysis also incorporated bottom temperature variables (tempmin_bdmin, tempmax_bdmax, tempmean_bdmean), which showed varying degrees of coupling with surface temperatures depending on water depth and season. Mean salinity remained consistently high (range: 37.04–37.46‰; mean = 37.20‰, SD = 0.08‰), with even small variations potentially ecologically significant in this hypersaline environment.

The shallow nature of the Gulf ensures extensive photic zone coverage, influencing productivity and oxygenation. Chlorophyll-*a* concentrations indicated predominantly oligotrophic conditions with localized high-productivity areas (range: 0.73–12.99 mg/m³; mean = 1.68 mg/m³, SD = 1.09 mg/m³; median = 1.27 mg/m³). Dissolved oxygen levels remained relatively high and stable (range: 4.51–5.02 ml/l; mean = 4.80 ml/l, SD = 0.11 ml/l). Nutrient concentrations varied across the gulf: silicate (range: 1.80–2.79 μmol/l; mean = 2.06 μmol/l, SD = 0.19 μmol/l), phosphate (range: 0.15–0.42 μmol/l; mean = 0.24 μmol/l, SD = 0.06 μmol/l), and nitrate (range: 0.81–2.58 μmol/l; mean = 1.66 μmol/l, SD = 0.43 μmol/l). pH showed minimal variation (range: 8.13–8.14; mean = 8.13, SD = 1.20e-3), while calcite concentrations widely fluctuated (range: 1.61e-4 − 5.60e-2 mol/m³; mean = 6.09e-3 mol/m³, SD = 1.12e-2 mol/m³).

Correlation analysis revealed significant relationships among environmental parameters. Notably, bathymetry was negatively correlated with salinity (r = −0.49) and minimum sea surface temperature (r = −0.46). Chlorophyll-*a* concentration was strongly correlated with the diffuse attenuation coefficient (r = 0.95), linking productivity and water clarity. Mean sea surface temperature showed strong positive correlations with nitrate concentration (r = 0.94), suggesting complex temperature-nutrient dynamics. Detailed maps visualizing the spatial distribution of major environmental parameters analyzed in this study are provided in supplementary materials (**10.5281/zenodo.15380710)**.

### Diversity of foraminifera

A total of 589 species of benthic foraminifera were collated from for the gulf, among these only taxa identified at species level were included for our modelling routine. In addition, occurrences that were not in the extent of the environmental variables and which did not meet stringent requirement for our modelling (i.e., species with three or less occurrences after spatial thinning) were removed. The model input dataset includes nine orders, 39 superfamilies, 89 families, 150 genera and 492 species. The distribution of diversity across these taxa was notably uneven, with two orders dominating the assemblage. Rotaliida exhibited the highest diversity, accounting for 54.9% of the total species count (270 species) and encompassing 39.3% of all families (35) and 44.0% of all genera (66) in the dataset. Rotaliida included important genera such as *Ammonia*, *Elphidium*, and *Bolivina*, which are known for their adaptability to various marine environments and their value as bioindicators. Miliolida ranked second, comprising 28.9% of the species (142) from 14.6% of families (13) and 30.7% of genera (46). Lituolida significantly contributed to the diversity with 32 species (6.5%), 11 families and 15 genera. Textulariida showed moderate diversity with 22 species (4.5%) from three families and three genera. Although less diverse than the dominant orders, Textulariida represented an important component of the agglutinated foraminiferal fauna in the region. The remaining five orders – Nodosariida (11 species), Polymorphinida (11 species), Astrorhizida (one species), Robertinida (one species), and Spirillinida (two species) – collectively represent 5.2% of the total species diversity. At the genus level, several taxa stood out for their exceptional species richness. In Miliolida, *Quinqueloculina* emerged as the most diverse genus, containing 69 species (14.0% of total species). This genus is known for its ability to thrive in various shallow marine environments. *Triloculina* followed with 26 species (5.3%), and *Spiroloculina* with 22 species (4.5%), both showcasing the diversity within miliolid morphologies. Within Rotaliida, *Elphidium* comprised 20 species (4.1%), reflecting its importance in shallow marine ecosystems. *Ammonia*, a genus commonly associated with stressed or transitional environments, included 15 species (3.0%). *Bolivina*, with 14 species (2.8%), represented an important component of the benthic foraminiferal assemblage, commonly indicative of high organic matter flux. The family Hauerinidae (Miliolida) emerged as the most diverse, with 24 genera and 116 species, highlighting the success of this family in the gulf environment. This family’s dominance may suggest high endemism and favourable conditions for porcelaneous foraminifera with complex chamber arrangements. Hyaline forms, corresponding largely to Rotaliida, represent 58.5% of the species, porcelaneous forms accounted for 28.9% of the species, while agglutinated forms, including Textulariida, Lituolida, and other orders, made up the remaining 12.6% of the species.

## Modelled data

### Species distribution models

Individual SDMs were evaluated using multiple metrics. The AUC values for CTA, RF, and SVM were 0.903, 0.952, and 0.950, respectively (Table 1), indicating excellent model performance across all algorithms. RF showed the highest predictive power, followed closely by SVM and then CTA. The sensitivity (true positive rate) ranged from 0.912 for RF to 0.922 for CTA, while specificity (true negative rate) ranged from 0.879 for CTA to 0.914 for RF. These high values suggested that the models were highly effective in correctly predicting both presence and absence of species across the study area. The Kappa statistic ranged from 0.801 for CTA to 0.827 for RF, indicating substantial agreement. The omission rates were notably low across all algorithms (CTA: 0.099, RF: 0.086, SVM: 0.089), further underscoring the models’ robust performance in capturing species distributions.

**Table 1:** Performance metrics of individual Species Distribution Models (SDMs) for Classification Tree Analysis (CTA), Random Forests (RF), and Support Vector Machines (SVM).

Algorithm	AUC	Omission. Rate	sensitivity	specificity	Prop. Correct	Kappa
CTA	0.91	0.10	0.92	0.90	0.87	0.80
RF	0.95	0.09	0.91	0.91	0.91	0.83
SVM	0.95	0.09	0.91	0.91	0.91	0.82

### Stacked SDM performance

The stacked SDM showed robust performance in predicting species richness patterns. We evaluated several ensemble combinations, all of which achieved a perfect Kappa score of 1 (Table 2). Among these top-performing ensembles, we selected the CTA-RF-SVM combination based on its overall superior performance across multiple indices. The chosen CTA-RF-SVM ensemble demonstrated a mean prediction success of 71.97% (SD = 5.57%), indicating that the model correctly predicted species presence or absence in nearly 72% of all cases across the entire assemblage. The mean sensitivity of 0.95 (SD = 0.11) suggests that the model was particularly effective at predicting species presence, while the mean specificity of 0.91 (SD = 0.08) indicated strong performance in predicting species absence. The mean Jaccard index of 0.48 (SD = 0.24) for the CTA-RF-SVM ensemble implied moderate to good compositional similarity between predicted and observed species assemblages. This was the highest Jaccard index among all the top-performing ensembles, further justifying our selection. The species richness error (24.51 ± 22.59 SD) suggested some discrepancy between predicted and observed species numbers, which was consistent with the challenges in modelling hyperdiverse communities. While other ensembles showed strengths in certain areas – for instance, the CTA-GBM-SVM ensemble had a lower species richness error (15.40 ± 17.35) – the CTA-RF-SVM ensemble provided the best balance across all metrics. It maintained high sensitivity without losing specificity, and achieved the highest Jaccard index, indicating better overall prediction of community composition.

**Table 2:** Comparative performance of top-performing ensemble Species Distribution Models showing mean ± standard deviation for key evaluation metrics.

Ensemble	Species Richness Error	Prediction Success	kappa	specificity	sensitivity	Jaccard
CTA-RF-SVM	24.51 ± 22.59	71.97 ± 5.57	1 ± 0	0.91 ± 0.08	0.95 ± 0.11	0.48 ± 0.24
CTA-GBM-SVM	15.40 ± 17.35	71.79 ± 4.39	1 ± 0	0.93 ± 0.06	0.65 ± 0.24	0.33 ± 0.17
GLM-RF-SVM	34.90 ± 28.52	69.22 ± 7.15	1 ± 0	0.88 ± 0.10	0.93 ± 0.13	0.40 ± 0.23
CTA-GAM-RF-SVM	24.77 ± 22.49	71.82 ± 5.62	1 ± 0	0.91 ± 0.08	0.94 ± 0.12	0.47 ± 0.24
CTA-GBM-MARS-RF-SVM	20.4 ± 18.45	71.59 ± 4.81	1 ± 0	0.92 ± 0.06	0.78 ± 0.19	0.38 ± 0.18

### Algorithm correlation

The correlation between algorithms in the retained stacked ensemble model was strong. CTA and RF showed a correlation of 0.863, CTA and SVM 0.721, and RF and SVM the strongest correlation at 0.860. These correlations suggested substantial agreement in the predictions of the different algorithms, particularly between RF and the other two methods, while each still contributing some unique information to the ensemble. This high level of agreement among different modelling approaches provided additional confidence in the robustness of our predictions of benthic foraminiferal distributions in the Arabian Gulf. The high agreement between different algorithms and strong performance metrics delivered confidence in the model’s ability to capture complex spatial patterns of foraminiferal diversity in this region, offering a solid foundation for further ecological insights.

### Regional diversity patterns

We divided the gulf into five distinct regions (i.e., Central, Northern, Southern, Eastern, and Western) for an easy comparison of the various habitats and observed species diversity patterns. Statistical analysis demonstrated highly significant regional differences in foraminiferal diversity (ANOVA, F.4,80821 = 68563*, p* < 2e-16). Post-hoc Tukey HSD tests confirmed that all pairwise comparisons between regions were significant (*p* < 0.001 for all comparisons).

**Fig 3:**
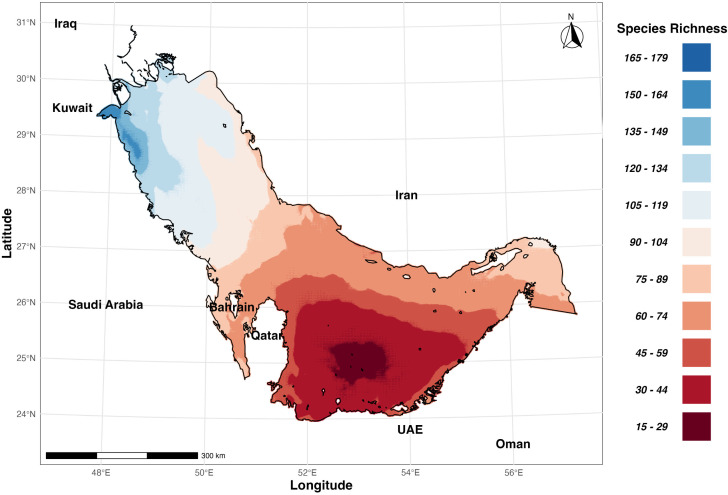
Interpolated map of foraminiferal species richness across the Arabian Gulf, highlighting diversity hotspots and their relationship to key environmental variables.

The Northern Gulf emerged as a diversity hotspot (Fig 3), exhibiting the highest mean diversity that significantly exceeded all other regions. The Northern Gulf hosted, on average, 65 more species than the Central Gulf (95% CI: 64.21–65.04). The Western Gulf showed the second-highest diversity levels, supporting an average of 40 more species than the Central Gulf (95% CI: 39.45–40.36). The Eastern Gulf displayed the third-highest diversity, with a mean increase of 15 species compared to the Central Gulf (95% CI: 13.90–14.80). The Central Gulf exhibited moderate diversity, serving as a baseline for inter-regional comparisons, while the Southern Gulf held markedly reduced diversity in this region, with an average of 19 fewer species compared to the Central Gulf (95% CI: 18.48–19.47), particularly the region between southern Qatar and northern coastline of UAE.

### Diversity gradients

Our analysis unveiled two primary diversity gradients (i.e., North-South Gradient and East-West Gradient), offering insights into the spatial organization of foraminiferal communities. A pronounced latitudinal gradient “North-South Gradient” emerged as the dominant pattern, with species richness increasing significantly from south to north (β = 18.69, t = 519.1, *p* < 2e-16). This robust relationship (R² = 0.7693, F1,80824 = 269500, p < 2e-16) underscored how the basin’s geographic configuration creates a strong north-south environmental gradient that shapes foraminiferal communities. A secondary, yet significant, east-west gradient was also observed, with diversity decreasing from east to west (β = −9.61, t = −242.4, p < 2e-16). This pattern accounted for 42.1% of the variance in diversity (R² = 0.421, F1,80824 = 58770, p < 2e-16), likely reflecting the basin’s asymmetric bathymetric profile and circulation pattern. This east-west pattern represented another basin-specific environmental gradient rather than a general longitudinal effect.

Moran’s (I) test revealed an exceptionally strong positive spatial autocorrelation in species richness (I = 1.000117, p < 2.2e-16), indicating that proximate locations host remarkably similar diversity levels. This spatial structure further supports our interpretation that foraminiferal diversity in the Arabian Gulf is primarily organized along basin-specific environmental gradients that reflect the unique geographical configuration and hydrodynamic patterns of this semi-enclosed sea, rather than broader biogeographic principles that might apply in open ocean systems. (Fig 3).

### Ensemble variable importance

**Fig 4:**
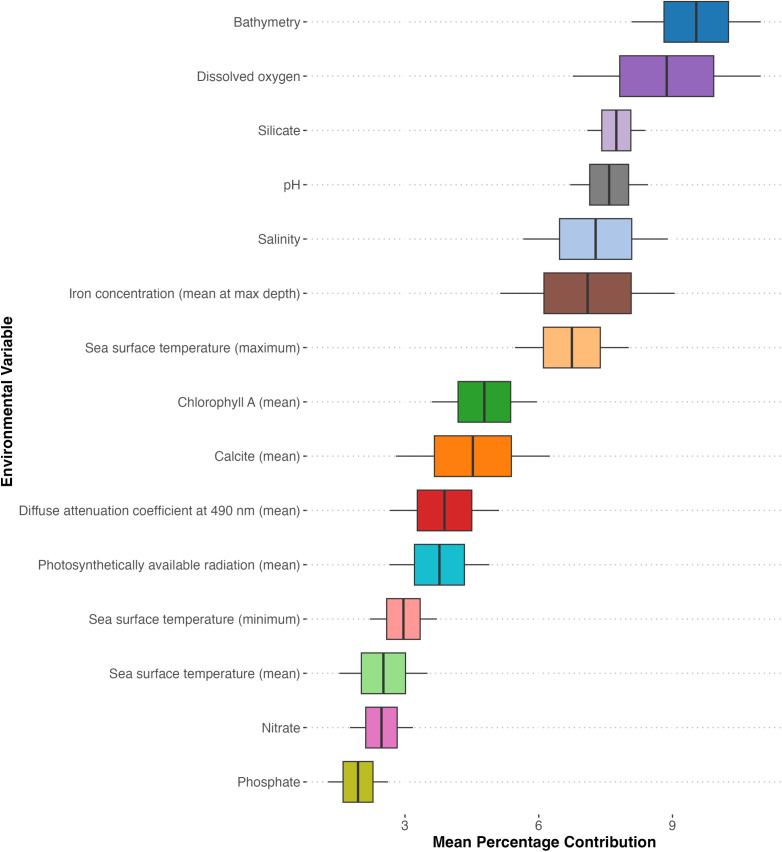
Relative importance of environmental predictors in the stacked species distribution model.

Our analysis of environmental variables revealed a complex hierarchy of factors influencing foraminiferal distributions in the Arabian Gulf (Fig 4). Bathymetry emerged as the primary driver, with a mean importance of 10.50% (SD = 6.29%). Dissolved oxygen closely followed bathymetry in importance (8.55 ± 3.30%), highlighting the crucial role of oxygen availability in foraminiferal ecology. Iron concentration ranked third in importance (9.17 ± 4.63%), surpassing traditional factors like salinity and temperature. Salinity, long recognized as a key factor in foraminiferal distributions, ranked fourth (8.64 ± 3.96%). Temperature effects showed a clear hierarchy, with maximum sea surface temperature (8.13 ± 4.00%) exerting greater influence than minimum (4.42 ± 1.85%) or mean (4.36 ± 1.48%) temperatures. Both pH (7.57 ± 4.01%) and silicate concentration (7.78 ± 4.32%) ranked highly in our models. Notably, macronutrients such as nitrate (3.72 ± 1.53%) and phosphate (3.51 ± 1.37%) had relatively low overall importance compared to other environmental variables.

**Fig 5:**
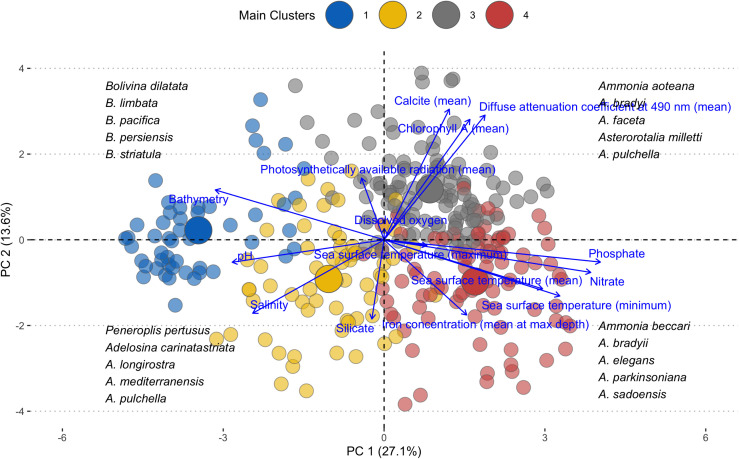
Hierarchical cluster analysis of principal components biplot with environmental clusters and associated foraminiferal assemblages.

**Fig 6:**
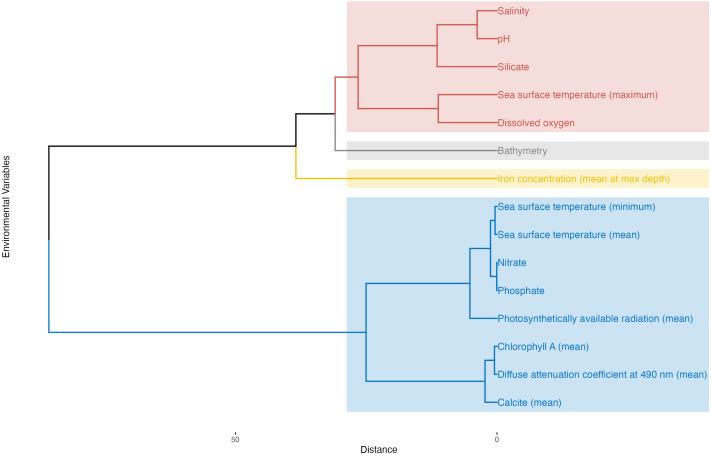
Dendrogram classification of environmental variables.

Building upon variable importance data, we used Hierarchical Cluster analysis of Principal Components (HCPC) to further characterize each group by examining representative species and assemblages. The analysis identified four distinct clusters (Figs. 5 and 6), each associated with specific environmental conditions and foraminiferal assemblages in the Gulf. **Cluster 1** was primarily defined by the importance of bathymetry, pH, salinity, and photosynthetically available radiation. Species strongly associated with this cluster included several *Bolivina* species (*B. dilatata, B. limbata, B. pacifica, B. persiensis,* and *B. striatula*), as well as *Trochammina advena, Cancris indicus, Alliatina excentrica, Ammobaculites persicus, Ammoscalaria pseudospiralis, Arenoparella oceanica, Hyalinea balthica,* and *Quadrimorphina laevigata*. The presence of agglutinated, calcareous, and hyaline forms suggested a diverse assemblage adapted to specific gradients represented by these variables. **Cluster 2** was characterized by the significance of silicate levels and maximum sea surface temperature. *Peneroplis pertusus* emerged as a key species in this cluster, along with several *Adelosina* (*A. carinatastriata, A. longirostra, A. mediterranensis, A. pulchella*) and *Ammonia* species (*A. aberdoveyensis, A. beccarii, A. convexa, A. tepida*). The co-occurrence of larger benthic foraminifera (*Peneroplis*) with smaller benthic forms may indicate a complex community structure in a shallow environment. **Cluster 3** was distinguished by the prominence of chlorophyll-*a*, light attenuation coefficient, calcite, and phosphate as important variables. *Quinqueloculina subcuneata* was identified as a characteristic species of this cluster. Other species included *Adelosina echinata, A. partschi, Affinetrina planciana, Articulina alticostata, Asterigerina simplex*, and two *Asterorotalia* species (*A. milletti* and *A. pulchella*). This cluster likely represented clear, oligotrophic waters offshore around coral reefs, with some opportunistic taxa that may thrive in nutrient-rich coastal environments. **Cluster 4** was defined by the importance of nitrate, phosphate, sea surface temperature (mean and minimum), iron concentration, and dissolved oxygen. This cluster was associated with well-oxygenated and intermediate productivity environments. It included additional *Adelosina* species (*A. crassicarinata, A. dagornae, A. intricata*), *Ammobaculites* agglutinans, and was particularly enriched in *Ammonia* species (*A. beccarii, A. bradyii, A. elegans, A. parkinsoniana, A. sadoensis,* and *A. umbonata*).

The recurring presence of certain genera across multiple clusters, particularly *Adelosina* and *Ammonia*, underscored their ecological flexibility and importance in this ecosystem. The *Ammonia* assemblage, well-represented in Cluster 4 but present across multiple clusters, suggested high adaptability and success in the Arabian Gulf. Similarly, the *Adelosina* assemblage, present in Clusters 2, 3, and 4, indicated ecological plasticity and adaptation to various niches within the gulf. The *Bolivina* assemblage, primarily in Cluster 1, appeared to be associated with deeper, more saline waters with higher pH. The *Peneroplis* assemblage in Cluster 2 was notable for its adaptation to environments with high silicate concentrations and maximum sea surface temperatures, typical of shallow-water, warm-adapted species in areas with constant terrigenous sand inputs. Lastly, the recurring presence of genera such as *Quinqueloculina, Elphidium,* and *Triloculina* across multiple clusters, while not forming distinct assemblages themselves, might indicate their adaptability to various ecosystems in the gulf.

## Discussions

### Model performance and methodological considerations

Our SSDM approach represents a significant advancement in mapping foraminiferal diversity at a basin-wide scale. The high performance of our models (mean AUC > 0.94, TSS > 0.8, Kappa > 0.82) demonstrates the power of this approach in capturing complex species-environment relationships in the challenging setting of a marginal sea. By combining individual species models, SSDMs provide a more comprehensive view of community-level diversity patterns, which is particularly valuable for benthic foraminifera where species interactions and community structure play crucial roles in their distribution and ecology [6].

The ensemble approach used in our SSDM, combining Classification Tree Analysis (CTA), Random Forests (RF), and Support Vector Machines (SVM), allows for more robust predictions than single-algorithm SDMs. This is evidenced by the high-performance metrics across all algorithms (AUC > 0.91, sensitivity ranging from 0.91 to 0.92, specificity from 0.90 to 0.91). The strong correlation between algorithms (CTA-RF: 0.863, CTA-SVM: 0.721, RF-SVM: 0.860) suggests substantial agreement in predictions while still contributing unique information to the ensemble. This multi-algorithm approach helps to mitigate the biases and limitations inherent in individual modelling techniques [80]. The strong spatial autocorrelation in species richness (Moran’s I = 1.000117, *p* < 2.2e-16) observed in our study highlights the importance of accounting for spatial dependencies in biodiversity modelling. SSDMs inherently capture some of these spatial patterns by combining multiple species models, providing a more realistic representation of biodiversity patterns [41,45].

### Spatial patterns of foraminiferal diversity

Our results demonstrate a clear north-south gradient in foraminiferal species richness (Fig 3), with the highest diversity observed in the northern part of the gulf. This modeled gradient strongly aligns with findings from numerous studies conducted in the northern sectors, including Al-Shuaibi et al. [89], Al-Zamel et al. [90–92], Parker and Gischler [93], Al-Enezi [94,95], and Khader [96,97], all of which documented high foraminiferal diversity in the coastal waters of Kuwait and northern Gulf. This pattern contrasts with the typical latitudinal diversity gradients observed in many marine taxa globally, where diversity generally decreases from lower to higher latitudes [98]. The inverse gradient detected in our study (i.e., increasing diversity towards higher latitudes) is particularly noteworthy and reflects the basin’s specific geographic features rather than the global-scale latitudinal effects observed in open oceans. This pattern primarily results from the freshwater influence from the Shatt al-Arab delta in the north and increasing restriction, residence time, and salinity toward the south. The northern Gulf, influenced by the Shatt al-Arab delta formed by the confluence of the Tigris, Euphrates, and Karun rivers, experiences significant freshwater input, creating a complex estuarine environment. This cooler freshwater influx not only modulates salinity but also introduces terrigenous nutrients and sediments, fostering habitat heterogeneity [99–101]. The interaction between marine and freshwater environments creates a mosaic of microhabitats, favorable for a wider range of species and potentially explaining the higher foraminiferal diversity in this region [91].

Conversely, the southern Gulf, particularly between southern Qatar and the northern coastline of the UAE, exhibits lower foraminiferal diversity. This can be attributed to higher residence times of water and the very shallow, laterally extensive nature of the basin, compounded by higher evapotranspiration rates [63]. Our findings for this southern region, particularly the reduced diversity in the hypersaline waters of Salwa Bay, corroborate the observations of Amao et al. [102] and Murray’s pioneering studies in the hypersaline environments of Abu Dhabi [23,47,52]. The extreme salinity gradient, increasing from approximately 37 in the north to over 50 in some southern areas, likely plays a crucial role in shaping diversity patterns [103]. Many foraminiferal species may be unable to tolerate these hypersaline conditions, leading to reduced diversity in the southern regions [47]. The east-west gradient, with overall higher diversity in the eastern part of the gulf, corroborates findings from other studies in the region (e.g., Amao et al.[104]) and corresponds with patterns documented by Lutze [53–55] and Amao et al. [105] along the Iranian coast in the Eastern region, Amao et al. [106] in Bahrain, and comparisons with Western Gulf studies by Cherif et al. [57] and Amao et al. [104]. This pattern likely reflects the influence of the counter-clockwise circulation pattern in the Gulf, which affects propagule dispersal and nutrient distribution [107,108]. Additionally, the Iranian coast to the east receives turbid freshwater input from numerous rivers, and its relatively deeper bathymetry further contributes to environmental complexity and potentially more diverse sedimentary environments, providing a wider range of niches for foraminiferal assemblages [63]. The distribution of wall types in our models—with porcelaneous taxa dominating Southern and Western regions, hyaline forms prevalent in the Eastern region, and agglutinated forms reaching their peak representation in the Eastern region—closely matches the trends reported by Murray [10], and Amao et al. [18].

### Taxonomic composition and diversity patterns

Our model input dataset reveals a rich and diverse foraminiferal assemblage in the Gulf, comprising of over 492 species belonging to nine orders, 39 superfamilies, 89 families, and 150 genera. The dominance of Rotaliida (54.9% of species) and Miliolida (28.9% of species) aligns with the typical composition of shallow, warm-water foraminiferal assemblages globally (Debenay, 2012). However, the exceptionally high diversity within these orders, particularly the abundance of *Quinqueloculina* (14.0% of total species), *Triloculina* (5.3%), and *Elphidium* (4.1%), surpasses those reported in many other marginal seas [109]. The prevalence of miliolid genera across multiple environmental clusters underscores their ecological flexibility. This adaptability, particularly to salinity fluctuations, makes miliolids valuable environmental indicators in the gulf [110]. Their porcelaneous tests, which are less susceptible to dissolution than hyaline forms, may provide more reliable long-term records of environmental change in this region characterized by high carbonate saturation states [111]. The high diversity of *Elphidium* species (4.1% of total species) is particularly noteworthy. *Elphidium* is known for its adaptability to a wide range of shallow marine environments and its sensitivity to environmental parameters such as salinity and organic matter input [10]. The abundance of *Elphidium* species in the gulf suggests a wide range of coastal and shelf habitats, potentially reflecting the complex interplay of freshwater influence, anthropogenic inputs, localized productivity, and hydrodynamic regimes across the basin.

The distribution of diversity across taxa is highly skewed, with Rotaliida and Miliolida together accounting for 83.8% of all species. This dominance suggests that these orders are particularly well-adapted to the extreme conditions of the Arabian Gulf. The family Hauerinidae (Miliolida) emerges as the most diverse, with 24 genera and 116 species, highlighting the success of this family in the Gulf environment. This family’s dominance suggests high endemism and favourable conditions for porcelaneous foraminifera with complex chamber arrangements. The presence of agglutinated forms, including Textulariida and Lituolida, albeit in lower proportions (12.6% of species), indicates the availability of suitable habitats for these taxa, possibly in areas with higher terrigenous input or specific substrate types. The relative scarcity of some orders (e.g., Spirillinida, Robertinida) is consistent with their generally low representation in most foraminiferal assemblages globally [10,112].

### Environmental drivers and niche partitioning

Our models identify bathymetry and dissolved oxygen as primary predictors of foraminiferal distributions (Fig 5), aligning with some of the TROX (TRophic OXygen) model parameters proposed by Jorissen et al. [28]. Bathymetry emerged as the most important statistical predictor (mean importance 10.50%, SD = 6.29%), though we recognize that depth itself is not a direct ecological driver for benthic foraminifera [32,113,114]. Rather, bathymetry in our models likely serves as a proxy for several depth-correlated factors, particularly light penetration which affects symbiont-bearing larger benthic foraminifera abundant in the Gulf (Fig 6). Additionally, in this shallow basin, bathymetry correlates with substrate type, hydrodynamic energy, temperature stability, and food availability, collectively creating depth-related microhabitat zonation. Dissolved oxygen follows closely in importance (8.55%, SD = 3.30%). In the shallow, warm, and mostly oligotrophic waters of the Gulf, the interplay between food availability and oxygen concentrations, both strongly influenced by water depth, appears to be a key factor in foraminiferal microhabitat selection and distribution. The importance of these factors in structuring foraminiferal communities has been documented across diverse marine environments globally, from continental shelves to deep-sea settings, suggesting fundamental constraints on foraminiferal distributions that transcend regional boundaries. While our analysis identifies bathymetry, iron concentration, salinity, and dissolved oxygen as the primary predictors of foraminiferal distributions when averaged across all species, it is crucial to recognize that this hierarchical ranking mask considerable species-specific variation in environmental responses. The mean importance values across environmental variables range from approximately 3% to 9%, indicating relatively subtle differences in overall influence rather than dominance by any single factor (Fig 4). This pattern reflects the complex environmental mosaic of the Gulf and highlights the ecological specialization within foraminiferal communities.

The species-specific analysis of foraminiferal taxa reveals that variables with lower mean importance commonly exert primary control on individual species distributions. For instance, while maximum sea surface temperature ranked fourth in overall importance, it emerges as the dominant control for several species, particularly those found in the shallow, warmer southern regions. Similarly, silicate concentration, though ranking fifth overall, proves critical for certain miliolid taxa associated with specific substrate types. This differential sensitivity to environmental parameters explains how such high foraminiferal diversity can persist within a basin characterized by extreme conditions.

The strength of our SSDM approach lies precisely in its ability to capture these nuanced, species-specific responses to environmental gradients. Traditional community-level approaches might identify only the broadly important variables, potentially obscuring the ecological processes that structure foraminiferal assemblages at finer scales. By first modeling each species individually and then aggregating the results, we capture both the general patterns and the species-specific exceptions that contribute to the complex spatial distribution of foraminifera across the gulf. The importance of iron concentration in our model, ranking third in variable importance (9.17%, SD = 4.63%), represents a significant contribution to foraminiferal ecology that extends beyond regional significance. While iron is known to be a limiting nutrient in many marine systems, its specific role in benthic foraminiferal ecology has been underexplored globally. Our findings align with emerging research indicating iron’s importance for symbiont-bearing larger benthic foraminifera [115], suggesting an important link that may apply to foraminiferal communities worldwide. Recent studies have identified iron as crucial for photosynthetic efficiency in endosymbiotic algae and for biomineralization processes in calcifying organisms [116,117]. The identification of iron as a key driver in the Gulf may offer new perspectives for understanding foraminiferal distributions in other iron-limited marine environments and provides a foundation for investigating this relationship in diverse oceanographic settings.

Salinity, long recognized as a key factor in foraminiferal distributions, ranks fourth (8.64%, SD = 3.96%). Its high importance reflects the unique hydrological conditions of the Gulf, characterized by high evaporation rates and variable freshwater inputs, creating a range of salinity regimes that influence species distribution. The varying importance of environmental variables among species highlights the fine-scale niche partitioning within the foraminiferal community. For instance, the high sensitivity of *H. balthica* to bathymetry corroborates its known preference for outer shelf and upper slope environments [118], suggesting it may be a useful stenobathic indicator in Gulf sediments and in analogous settings globally. The strong association of *A. beccarii* with dissolved oxygen levels reflects its adaptation to fluctuating coastal conditions and its potential as a bioindicator of oxygen availability [119], a relationship documented across diverse coastal environments worldwide. Hierarchical clustering (Fig 5) of the species based on their environmental responses identified distinct ecological groups with different primary environmental controls. This fine-scale niche partitioning is evident in our HCPC analysis, where species show varied contributions to different environmental axes. For example, species within the *Ammonia* complex demonstrate greater sensitivity to variables associated with coastal environments (salinity fluctuation, nutrient input), while deeper-water taxa in the *Bolivina* group respond more strongly to bathymetry and oxygen availability. The identification of these species-specific environmental thresholds provides critical information for interpreting foraminiferal assemblages in both modern monitoring efforts and paleoenvironmental reconstructions.

The high ranking of pH in our models suggests that foraminiferal communities in the gulf may be sensitive to variations in ocean acidification. However, as our study relies on compiled occurrence data rather than physical specimen examination, we cannot directly confirm pH effects on shell preservation or morphology. Engel et al. [120] have demonstrated that larger benthic foraminifera exposed to pH extremes show significant shell dissolution, with species-specific vulnerability patterns that could influence community composition. In the Gulf context, where high temperatures and salinity already create physiological stress, pH variations may represent an additional stressor that influences species distributions. The relatively low importance of macronutrients such as nitrate and phosphate compared to other environmental parameters aligns with previous observations that many foraminifera in carbonate-dominated, oligotrophic settings like the Gulf may be adapted to low-nutrient conditions. This is particularly true for symbiont-bearing species that can thrive under nutrient limitation through their symbiotic relationships with algae.

Temperature effects show a clear hierarchy, with maximum sea surface temperature (8.13%, SD = 4.00%) exerting greater influence than minimum (4.42%, SD = 1.85%) or mean (4.36%, SD = 1.48%) temperatures. This pattern suggests that extreme thermal maxima may be more ecologically relevant than average conditions, potentially indicating thermal stress as a key factor in shaping foraminiferal communities in this warm, shallow basin. As global ocean temperatures continue to rise, this finding has significant implications for predicting how foraminiferal communities may respond to increasing thermal stress in other marine environments worldwide. The greater importance of maximum rather than mean temperatures aligns with the growing recognition that extreme events, rather than changes in average conditions, may be primary drivers of ecological responses to climate change. The identification of distinct foraminiferal assemblages associated with specific environmental clusters provides further insight into niche partitioning and community structure in the gulf. For example, the *Bolivina* assemblage is predominantly found in the deeper parts of the basin, which also coincide with more saline bottom waters in the Arabian Gulf. This distribution pattern may reflect stratification in these deeper areas, creating conditions that favour *Bolivina* species, which are globally known for their affinity to organic-rich, low-oxygen environments [13,29]. The abundance of *Bolivina* species in these settings may indicate periods of enhanced stratification or increased productivity in the Gulf’s history, possibly linked to variations in the inflow of oceanic waters through the Strait of Hormuz. Similar *Bolivina*-dominated assemblages have been documented in oxygen minimum zones worldwide, suggesting consistent ecological preferences that transcend regional boundaries [121].

The *Peneroplis*-dominated assemblage in areas with high silicate concentrations and maximum sea surface temperatures is particularly significant for understanding the Gulf’s carbonate budget. As important carbonate producers, the distribution of *Peneroplis* and other larger benthic foraminifera may help in reconstructing past sea surface temperatures and in quantifying carbonate production rates under different climatic regimes. This relationship between larger benthic foraminifera and carbonate production has global significance for understanding how reef-associated environments may respond to climate change. The areas of highest species richness in the northern Gulf correspond with regions where multiple environmental variables create heterogeneous microhabitats. This environmental complexity allows for the coexistence of species with different ecological requirements, supporting our finding that environmental heterogeneity drives diversity patterns in the basin. In addition, the subtle gradations in variable importance we observed suggest that foraminiferal communities in the Arabian Gulf respond to a complex interplay of environmental factors rather than being controlled by just one or two dominant variables. This ecological complexity underscores the value of multivariate approaches in understanding foraminiferal distribution patterns in extreme environments.

### Implications for ecology and climate change

Our findings may have profound implications for understanding the Gulf’s past and future ecology. The strong environmental control on foraminiferal distributions suggests that these communities may be particularly vulnerable to anthropogenic impacts and climate change. The projected increases in temperature and salinity in the Gulf [122] could lead to significant shifts in foraminiferal assemblages, potentially affecting carbonate production and sediment stability. The fact that our models can delineate each species niche and environmental gradient means we can now efficiently and proactively model species responses to changes in temperature or other extremes brought about by the increasing change in climate. Species sensitive to temperature fluctuations can now be selectively modelled and their responses properly documented, providing a powerful tool for predicting and monitoring ecosystem changes. The identification of diversity hotspots, particularly in the northern Gulf and along the Iranian coast, highlights areas of high conservation priority for marine organisms in general. These regions may serve as important refugia as environmental conditions in the Gulf become more extreme. However, these areas are also subject to intense human activity, including oil extraction, coastal development, and decreasing freshwater input from rivers, underscoring the need for targeted conservation efforts to preserve these unique marine communities.

While our presence/absence-based SDM approach provides valuable insights into spatial biodiversity patterns and environmental drivers, we acknowledge its limitations for environmental monitoring applications. Environmental changes, particularly in early stages, typically manifest as shifts in relative species abundances rather than immediate local extinctions or colonisations. Stress-tolerant taxa may persist but become numerically dominant as conditions deteriorate, while sensitive species may decline in abundance long before disappearing entirely. These abundance shifts, which can serve as early warning signals of ecosystem change, are not captured in binary presence/absence models. Future work could complement our approach by incorporating abundance data where available, potentially through techniques such as point process models or joint species distribution models that can accommodate both occurrence and abundance information. Such abundance-based extensions would be particularly valuable for establishing monitoring protocols in vulnerable regions like the northern Gulf diversity hotspot, where early detection of community composition shifts could provide critical information about ecosystem responses to anthropogenic stressors. Nevertheless, our presence/absence models provide a solid foundation by identifying the fundamental environmental drivers and species-specific tolerances that ultimately determine which species can persist as conditions change.

## Conclusion

This basin-wide assessment of benthic foraminiferal diversity in the Arabian Gulf reveals complex spatial patterns and environmental relationships that may both inform regional ecology and contribute to broader understanding of biodiversity determinants in extreme environments. Our study documents a pronounced north-south diversity gradient that contrasts with typical latitudinal patterns observed in open marine systems, highlighting how local environmental filters can override global biodiversity trends in semi-enclosed basins. The inverse latitudinal diversity gradient, with highest species richness in the northern Gulf, demonstrates that environmental heterogeneity and stress gradients can be more influential in structuring foraminiferal metacommunities than latitude alone, a finding relevant to biodiversity theory more broadly. The methodological framework developed here; combining SSDMs with comprehensive environmental characterization, demonstrates exceptional performance in capturing species-environment relationships in a challenging setting. This approach transcends regional application, offering a template for similar assessments in other semi-enclosed seas and marginal marine environments worldwide. The ability to model individual species distributions and then aggregate them to examine community-level patterns provides a powerful tool for investigating diversity patterns across diverse taxonomic groups and environmental gradients beyond the Arabian Gulf.

Our identification of key environmental drivers shaping foraminiferal distributions contributes to fundamental understanding of foraminiferal autecology. While bathymetry and dissolved oxygen emerged as primary determinants when averaged across all species, our analysis reveals considerable variation in environmental responses among individual taxa. This ecological specialization, where different species respond most strongly to different environmental variables, explains how such high foraminiferal diversity can persist despite the extreme conditions of the Arabian Gulf. The unexpected importance of iron concentration represents a novel finding with potential implications for foraminiferal distributions in other iron-limited marine systems. The hierarchical importance of temperature variables—with thermal maxima exerting greater influence than means, may provide insights into how thermal stress may shape foraminiferal communities under future warming scenarios across tropical and subtropical seas globally. The delineation of species-specific fundamental niches achieved through our modeling approach enables prediction of community responses to environmental change at unprecedented resolution. This capability extends beyond regional significance, offering a mechanism for forecasting how foraminiferal assemblages in diverse marine settings may respond to climate-driven environmental changes. As marine ecosystems worldwide face increasing thermal stress, salinity fluctuations, and deoxygenation, the species-environment relationships quantified here provide reference points for anticipating community shifts and identifying potential indicator taxa across ecotones.

For paleoenvironmental applications, our findings may be used to refine understanding of the environmental preferences of key foraminiferal taxa, potentially improving the interpretation of fossil assemblages from the Arabian Gulf and analogous settings globally. The strong associations between specific genera and environmental parameters documented here can strengthen the foundation for foraminiferal-based paleoenvironmental reconstructions, enhancing their utility for understanding past environmental conditions across diverse depositional environments. As anthropogenic impacts and climate change continue to alter marine ecosystems, the approach and results presented here will be instrumental in predicting and monitoring the responses of these ecologically important organisms to environmental perturbations. The Arabian Gulf, with its extreme environmental conditions, serves as a natural mesocosm for understanding how marine communities may function under future climate scenarios, providing insights that extend well beyond this region to inform global understanding of biodiversity patterns and ecological processes in marginal seas worldwide.
